# Physical activity in pregnancy: a mixed methods process evaluation of the FitMum randomised controlled trial interventions

**DOI:** 10.1186/s12889-022-14717-1

**Published:** 2022-12-06

**Authors:** Signe de Place Knudsen, Caroline Borup Roland, Saud Abdulaziz Alomairah, Anne Dsane Jessen, Stig Molsted, Tine D. Clausen, Ellen Løkkegaard, Bente Stallknecht, Julie Bønnelycke, Jane M. Bendix, Helle Terkildsen Maindal

**Affiliations:** 1 Department of Gynaecology and Obstetrics, Copenhagen University Hospital - North Zealand, Hillerod, Denmark; 2 Department of Biomedical Sciences, University of Copenhagen, Copenhagen, Denmark; 3 Department of Public Health, College of Health Sciences, Saudi Electronic University, Riyadh, Saudi Arabia; 4 Department of Clinical Research, Copenhagen University Hospital - North Zealand, Hillerod, Denmark; 5 Department of Clinical Medicine, University of Copenhagen, Copenhagen, Denmark; 6 Department of Visual Culture and Performance Design, Roskilde University, Roskilde, Denmark; 7 Department of Public Health, Aarhus University, Aarhus, Denmark; 8 Steno Diabetes Center Copenhagen, Herlev, Denmark

**Keywords:** Complex interventions, Process evaluation, Mixed methods, Intervention research, Physical activity, Pregnancy

## Abstract

**Background:**

Physical activity (PA) at moderate intensity is recommended for healthy pregnant women. The three-arm FitMum randomised controlled trial showed that it was possible to increase PA level during pregnancy with structured supervised exercise training (EXE) compared to standard care. Motivational counselling on PA (MOT) did not increase PA. This process evaluation aims to understand the implementation and mechanisms of impact of EXE and MOT.

**Methods:**

A mixed methods process evaluation was conducted using the UK Medical Research Council’s process evaluation framework by assessing implementation (*reach, fidelity, and dose*) and *mechanisms of impact* of the two interventions provided to pregnant women in FitMum. Data was collected both quantitatively (*n* = 220) and qualitatively (*n* = 20).

**Results:**

The FitMum trial *reached* educated pregnant women (80% having an educational level ≥ bachelor’s degree) with high autonomy of everyday life. Most participants (58%) were recruited at their first-trimester ultrasonic scan. Reasons to participate were personal (91%) and altruistic (56%). The intervention *dose* was delivered as intended with high *fidelity* in the original physical intervention setup and in the altered online setup during the COVID-19 restrictions. A low *dose* received in EXE (1.3 [95% CI, 1.1; 1.5] sessions/week) was partly explained by the pre-scheduled EXE sessions favouring participants with a flexible everyday life and a supportive social network. *Dose* received in EXE increased during online intervention delivery. Participants in MOT received 5.2 [4.7; 5.7] of 7 sessions. *Mechanisms of impact* comprised a perception of intervention commitment among participants in EXE due to the scheduled EXE sessions, whereas participants in MOT considered themselves as PA self-determined. PA was considered as constrained activities in EXE and included in daily activities in MOT.

**Conclusion:**

The FitMum interventions was *delivered* with high *fidelity*. During COVID-19, the *dose* received in EXE increased compared to the previous physical setup. Mechanisms of impact as commitment, perception of empowerment and perception of PA as well as the paradox between prioritising PA and family and the need of a flexible everyday life need to be considered when offering pregnant women PA interventions. Future interventions should consider a combination of physical and online exercise training for pregnant women.

**Supplementary Information:**

The online version contains supplementary material available at 10.1186/s12889-022-14717-1.

## Background

Physical activity (PA) during pregnancy is a safe and effective way of reducing pregnancy related complications in healthy women including excessive gestational weight gain [1, 2], gestational diabetes mellitus [3, 4], gestational hypertension, pre-eclampsia [5, 6], preterm delivery, caesarean section [3, 4, 7, 8], and depression [9]. Most pregnant women believe that PA during pregnancy is important and beneficial [10]. Despite this, a large percentage of pregnant women do not achieve the PA levels during pregnancy, as advised by the official recommendations, and some pregnant women even decrease their PA level over the course of pregnancy [5]. Intrapersonal barriers including fatigue, lack of time and motivation, and pregnancy discomforts, are the most frequently reported factors related to low PA levels [10, 11]. In addition, some pregnant women feel uncertain about whether participation in exercise interventions might harm their unborn child [12].

Various intervention strategies have been tested to promote PA during pregnancy. However, few studies have reported any superior interventions for increasing PA [13, 14]. In addition, intervention adherence has varied, often with no or inconclusive explanations [15, 16]. Given the complexity of PA interventions and the need to evaluate their impact, there is a need to test the efficacy with various methods.

Our research group developed the FitMum trial to investigate the effect of receiving structured supervised exercise training (EXE) or motivational counselling on PA (MOT), compared to standard care (CON), on PA levels. EXE and MOT will hereafter be referred to as the interventions. All women, who were to give birth at Copenhagen University Hospital - North Zealand, were informed about the FitMum trial. Healthy, inactive pregnant women who met the inclusion criteria were enrolled in the trial [17]. The effect evaluation of the FitMum trial [18] showed that participants in EXE had a higher level of moderate-to-vigorous-intensity PA (MVPA) (min/week) compared to participants in CON. However, the mean MVPA level in EXE corresponded to one third of the internationally recommended level [19]. No effect on MVPA was found in MOT compared to CON, and MVPA did not differ significantly between MOT and EXE. To fully understand the interventions and avoid the simplification of essential details a process evaluation is needed to monitor and document the implementation of the interventions [20, 21]. In this way, it is possible to understand why an intervention was or was not successful and to uncover impact mechanisms behind the results achieved [20, 21]. However, despite its importance, only few studies have provided knowledge regarding the mechanisms behind successful prenatal PA interventions [22–24].

The UK Medical Research Council guideline for process evaluation from 2015 [20] suggests that both quantitative and qualitative methods are equally essential in process evaluations to examine if the intervention reached the audience as intended (*reach*), if components of the complex intervention were provided as intended (*fidelity*), the quantity and quality of what was actually implemented (*dose*) and how the interventions produced or prevented changes (*mechanisms of impact*).

This mixed methods process evaluation aims to improve the understanding of the effects on PA that emerged from the effect evaluation of the FitMum trial [18]. doi: 10.2196/37699) and to gain insight into factors influencing the interventions by assessing the implementation (*reach, fidelity, and dose*) and the *mechanisms of impact* of the two complex PA interventions delivered to pregnant women participating in the FitMum randomised controlled trial.

## Methods

### Trial design

The process evaluation of the FitMum trial adapted the UK Medical Research Council process evaluation framework developed by Moore et al. in 2015 [20]. The framework was applied to investigate implementation components of the two interventions delivered in the FitMum trial covering intervention *reach, fidelity, dose*, and *mechanisms of impact*. The process evaluation was nested inside the FitMum trial with a mixed methods intervention design applied to let the qualitative strands help interpret and contextualise the quantitative results [25].

**Fig. 1:**
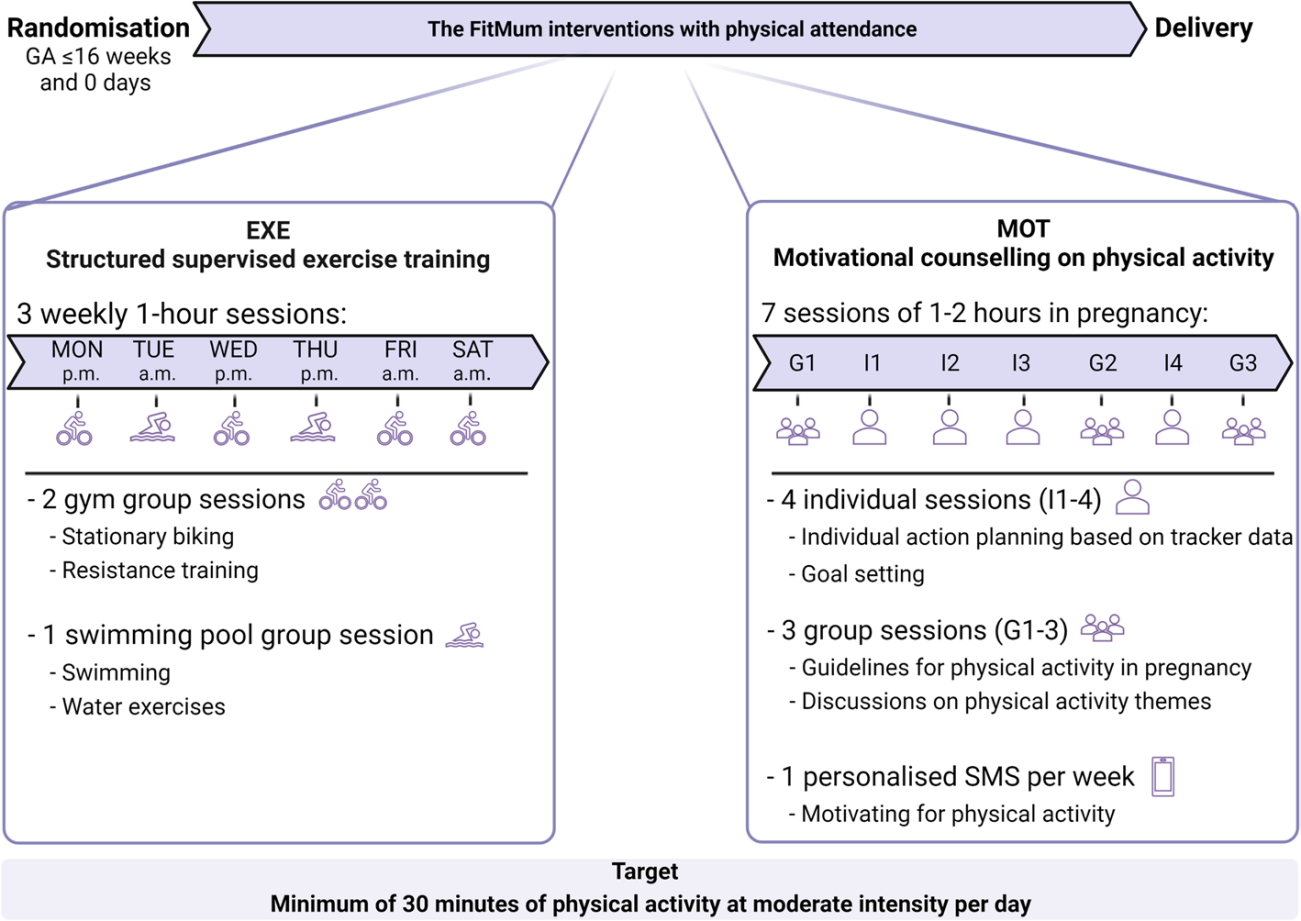
Content of the structured supervised exercise training intervention (EXE) and the motivational counselling on physical activity intervention (MOT) as they were designed originally. Mon, Monday; Tue, Tuesday; Wed, Wednesday; Thu, Thursday; Fri, Friday; Sat, Saturday; G, group counselling session; I, individual counselling session. The figure is created with Biorender.com

The FitMum trial was a single-site, three-arm randomised controlled trial that included 220 healthy inactive (less than one hour/week of MVPA during early pregnancy) pregnant women in a two-year period from October 2018 to October 2020. One participant was lost to follow-up before randomisation; hence 219 participants were randomised into CON (*n* = 45), EXE (*n* = 87), or MOT (*n* = 87). CON received standard care. The interventions (EXE and MOT) ran from randomisation to birth targeting a minimum 30 min/day of moderate intensity PA. The primary outcome of the interventions was MVPA in min/day. The content of the two interventions are illustrated in Fig. 1 and described in detail elsewhere [17]. In brief, the EXE group was offered 1-hour group-based supervised exercise training at moderate intensity 3 times/week in a gym and swimming pool. The MOT group was offered 4 individual and 3 group PA motivational counselling face-to-face sessions of 1 to 2 h duration during pregnancy and 1 weekly personalized text message to support PA. EXE sessions were offered six days/week and the participants in EXE were encouraged to choose three sessions/week. During the six days/week, gym sessions were offered four days (Mon, Wed, Fri and Sat). Swimming pool sessions were offered twice/week (Tue and Thu). Morning sessions were held three times/week (Tue 7:15 a.m., Fri 7:00 a.m. and Sat 9:00 a.m.); Afternoon sessions were held three times/week (Mon 4:30 p.m., Wed 4:30 p.m. and Thu 4:45 p.m.). Participants in MOT were offered seven sessions during the intervention period. Distribution of the seven counselling sessions in MOT: G1, < 3 weeks after randomisation; I1, 4–6 weeks after randomisation; I2 and I3, equally distributed between I1 and G2; G2, gestational age (GA) 24–26 weeks; I4, GA 31–32 weeks; G3, GA 35–37 weeks.

### Data collection and components

Data collection methods included quantitative data and semi-structured individual interviews of the participants. Table 1 presents the process evaluation dimensions, their definitions and corresponding measurements.

**Table 1:** Data collection of the process evaluation of structured supervised exercise training (EXE) and motivational counselling on physical activity (MOT). The dimensions addressed are in relation to implementation (reach, fidelity, and dose) and mechanisms of impact. Quantitative measures of reach were obtained at enrolment from all 220 included participants. Quantitative measures of fidelity, dose delivered and received, and mechanisms of impact were obtained during the intervention period from 87 participants randomised to each intervention group. All qualitative inquiries were obtained from 10 participants in EXE and 10 participants in MOT at the 35th gestational week

Process evaluation dimension	Definition	Quantitative measures	Qualitative inquiries
**Reach**	Participants included in the FitMum trial	Number of participants included in the FitMum trial and their reasons to participate	What were the reasons to participate?
**Fidelity**	To what extent the interventions were implemented as intended according to the protocol	How COVID-19 restrictions affected intervention implementation	Not obtained
**Dose delivered**	The number of intended intervention sessions conducted	How often the sessions were offered	How was intervention accessibility experienced?
			How did participants organise themselves in their everyday life to participate in the interventions?
**Dose received**	To what extent participants used resources as recommended	To what extent participants adhered to the interventions	What barriers and facilitators did the participants meet towards physical activity?
**Mechanisms of impact**	How the delivered interventions produced changes	Not obtained	How did the participants experience and perceive the impact of the interventions and what were their physical activity motives?

#### Quantitative measures

*Reach* was covered at inclusion by asking participants where they were introduced to the FitMum trial and their immediate reason(s) to participate. Answers were quantified into predefined options based on the recruitment strategy (online booking of ultrasonic scan, outpatient clinic at the hospital, posters at e.g. the general practitioner, social media, family and friends, online pregnancy related platforms or other options) and generally known reasons for participating in intervention studies (to increase PA, contribute to research, closer contact with health professionals, interact with peers or other reasons). *Fidelity* was assessed during the trial period comparing the FitMum trial protocol [17] with how intervention components were carried out, e.g. before and during COVID-19. *Dose* was assessed administratively by recording intervention attendance after each session from randomisation to giving birth.

#### Qualitative interviews

Semi-structured individual interviews [26] were conducted between July and December 2019 (before the COVID-19 restrictions) on a subset of enrolled participants during a test visit at the 35th gestational week. Of the 26 women approached, one woman cancelled the visit, and five women were prevented from participating in an interview for different reasons. An interview guide [26] with the following themes was used: Inclusion and participation in the FitMum trial, perception of the content in the interventions, PA in everyday life, barriers and enablers towards PA and the importance of PA during pregnancy (Supplementary file 1). *Reach* was assessed based on participants elaborating on their reasons for participating. *Dose* was evaluated based on participants giving insight to their everyday lives and the way they interacted with the interventions. *Mechanisms of impact* was covered based on the participants’ experience of the impact of the intervention components and key enablers and barriers that may influence the implementation and efficacy of the interventions [20].

All interviews were conducted by project staff at the FitMum trial facilities at Copenhagen University Hospital - North Zealand and lasted from 31 min to 1 h and 3 min, with an average of 48 min. All interviews were audio-recorded, added to the Research Electronic Data Capture (REDCap) and subsequently transcribed verbatim in their full length.

### Analysis

#### Integration of quantitative and qualitative data

Data analyses of quantitative and qualitative data were performed independently, and the findings were embedded within the mixed methods intervention design applied to let the qualitative strands help interpret and contextualise the quantitative results [25]. Quantitative and qualitative data were equally prioritised and presented theme-by-theme using a “weaving technique” reported in a narrative form [27]. Linkages between the quantitative and the qualitative findings led to three anticipated strategies: (1) confirmation, when results from quantitative and qualitative material confirmed each other, (2) expansion, when results of analyses of quantitative and qualitative data were different and extended insights occurred and (3) discrepancy, when results of quantitative and qualitative data were inconsistent and contradicted each other [27, 28].

#### Quantitative data to explore reach, fidelity and dose

Descriptive statistics of characteristics of the participants in the FitMum trial are presented as means ± standard deviation for symmetric distributions and medians (interquartile ranges) for skewed data. Categorical variables are presented as numbers (n) and frequencies (%). Wald-based 95% confidence intervals are given for reported intervention attendance estimates. Analysis regarding trial alterations due to the COVID-19 restrictions included participants who received either exclusively the physical intervention or exclusively the online intervention.

#### Qualitative analysis to explore reach, dose, and mechanisms of impact

A thematic content analysis of the interviews was performed using NVivo version 1.6.1 [29, 30] to identify patterns in data. First, SdPK (first author) obtained the total impression of the material by listening to all audio-recordings and reading all transcripts. Second, the interviews were coded separately on a line-by-line basis and initially organized according to the topic of questions from the interview guide in a systematic text condensation [30]. Codes were then inductively derived considering different intervention components and the dimensions of the evaluation framework. SdPK and JBø (co-author) discussed the coding structure, and issues were resolved by consensus. Third, SdPK and JBø developed themes to map each dimension of the framework. Identified themes were supported by direct quotes from the interviewees. The interview guide and all quotes involved in the manuscript were translated from Danish to English.

## Results

### Characteristics of participants

Two hundred and twenty healthy, inactive pregnant women were included in the FitMum trial and 219 with a gestational age of 12.9 (9.4–13.9) (median (IQR)) weeks were randomised (Table 2).

**Table 2:** Baseline characteristics of the participants in FitMum and the subset of participants who were interviewed. Descriptive data are presented as means ± SD for symmetrically distributions, medians (IQR) for skewed data, and n (%). School ≥ 12 years corresponds to high school. Further education ≥ 3 years corresponds to a university degree (bachelor or master level). No statistical comparisons have been performed on descriptive characteristics in accordance with CONSORT recommendations. SD, standard deviation; IQR, interquartile range; n, number; CON, control group; EXE, structured supervised exercise training; MOT, motivational counselling on physical activity

	Participants randomised to the FitMum trial				Interviewees		
	ALL	CON	EXE	MOT	All	EXE	MOT
	*n* = 219	*n* = 45	*n* = 87	*n* = 87	*n* = 20	*n* = 10	*n* = 10
Age (years), mean	31.5 ± 4.3	32.0 ± 4.6	31.1 ± 4.3	31.7 ± 4.1	32.3 ± 4.0	31.2 ± 3.4	33.3 ± 4.5
Gestational age at inclusion (weeks), median	12.9 (9.4–13.9)	12.9 (9.7–13.9)	12.6 (9.3–13.7)	12.9 (9.6–13.9)	11.3 (9.7–13.1)	11.5 (9.7–13.5)	11.2 (9.9–12.8)
Weight at inclusion (kg), mean	75.4 ± 15.3	72.0 ± 13.7	76.2 ± 17.4	76.3 ± 13.8	73.3 ± 17.1	72.7 ± 15.6	73.9 ± 19.4
Prepregnancy BMI (kg/m2), median	24.1 (21.8–28.7)	23.5 (21.3–26.8)	25.1 (21.5–29.7)	24.1 (22.4–28.9)	23.7 (21.5–28.1)	23.7 (21.8–27.3)	24.3 (21.3–30.3)
Nulliparity	82 (37)	16 (36)	40 (46)	26 (30)	8 (40)	4 (40)	4 (40)
Educational level							
School ≥ 12 years	191 (87)	41 (91)	74 (85)	76 (87)	18 (90)	9 (90)	9 (90)
Further education ≥ 3 years	175 (80)	33 (73)	73 (84)	69 (79)	18 (90)	9 (90)	9 (90)
Employed or studying	199 (91)	39 (87)	83 (95)	77 (89)	17 (85)	9 (90)	8 (80)

Of the randomised participants 80% had an educational level ≥ bachelor’s degree. A total of 20 interviews were conducted; 10 interviews of participants randomised to EXE or MOT, respectively (Table 2). Maternal baseline characteristics of the subset of 20 interviewees and the 219 randomised participants in the FitMum trial did not seem to differ.

### Reach

Of the included participants, 58% (*n* = 128) reported, that they were introduced to the FitMum trial while booking their first-trimester ultrasonic scan, 20% (*n* = 45) at the outpatient clinic at Copenhagen University Hospital - North Zealand, 15% (*n* = 32) via posters at e.g. their general practitioner, 10% (*n* = 23) via social media, 8% (*n* = 18) via friends or family, 5% (*n* = 12) via an online Danish pregnancy platform [31], and 9% (*n* = 19) via other options. Before randomisation, 91% (*n* = 201) stated that they wanted to participate in the trial to increase their level of PA, 56% (*n* = 123) to take part in and contribute to research, 7% (*n* = 16) to have a closer contact with health professionals, 5% (*n* = 10) to interact with other pregnant women, and 8% (*n* = 18) had other reasons. Participants in both intervention groups expressed in the interviews that the desire to become more physically active was mostly for the woman’s own good and arose from various factors; in general, there was an underlying understanding that the body naturally weakens during pregnancy. Hence, a physically active pregnancy was equated to an uncomplicated pregnancy with e.g., less pain and decreased risk of pregnancy complications. In extension, the participants reasoned that an uncomplicated pregnancy would lead to an uncomplicated delivery and emphasised that being in a good physical condition was a prerequisite for an uncomplicated delivery. The women assumed that their PA level would be low and mainly reserved to general everyday activities if not being a part of the interventions. One woman linked a hypothetically low PA level with self-blame and expressed that:

*“(If not being a part of the intervention) I could fear that I was still on the couch at home. That I hadn’t gotten my act together. And then I think I would have felt guilty if I then had an awful delivery. I could blame myself a bit for that, actually”* (Participant no. 117, EXE).

It appeared that the desire to become more physically active unconsciously resulted in a feeling of responsibility not only for the woman herself, but also in terms of the birth outcome and the well-being of the child. In addition, excessive gestational weight gain was framed as a concern. Some women stated that they had gained more weight than wanted in their previous pregnancies and by being physically active they wanted to limit their weight gain in their present pregnancy. One woman explained that she, because of being overweight, felt a greater responsibility to be physically active during the pregnancy. She expressed a concern about being judged by others if she did not try to improve the health of her unborn child through PA.

### Fidelity

The original planned sessions were held for 17.5 months with 120 participants included (CON: *n* = 24, EXE: *n* = 48, MOT: *n* = 48). In this period participants in EXE and MOT received physical interventions only. On March 11th, 2020 COVID-19 restrictions were implemented in Denmark. Thus, the original setup of EXE and MOT with physical attendance was altered into an online design of both interventions with participants attending from home [17]. Overall, the online interventions applied the same conditions as the physical interventions. However, in the online setup, EXE sessions were held virtually with 30 min of individual, offline, and self-selected aerobic PA followed by 30 min online structured aerobic and resistance training in groups (except for three months with pertained authority that allowed swimming pool access). In MOT, the content and distribution of group and individual sessions remained the same, however held online.

The online sessions ran for 14.5 months with 63 participants (CON: *n* = 14, EXE: *n* = 25, MOT: *n* = 24). Participants in EXE and MOT received the online interventions only as they were included and gave birth during the pandemic. Thirty-six participants (CON: *n* = 7, EXE: *n* = 14, MOT: *n* = 15) were included before the COVID-19 restrictions but gave birth during the pandemic. Participants in EXE and MOT received both the physical and online intervention. There were no differences in the lost to follow-up rate between participants who were included before or during COVID-19 restrictions.

### Dose delivered

**Fig. 2:**
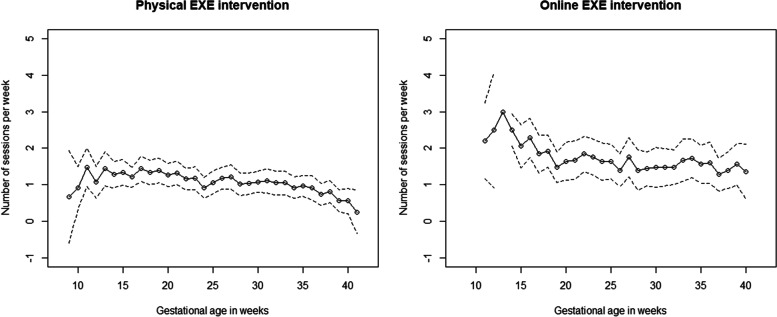
The average weekly number of structured supervised exercise training (EXE) sessions attended in the physical (left) and online (right) interventions, respectively. All participants randomised to EXE (*n* = 87) are included. The attendance was registered from randomisation (~ gestational age 10) to birth (~ gestational week 40). Full line, mean number of sessions attended; Dotted lines, 95% confidence interval. The confidence interval at gestational week 13 in the right plot (online interventions) was not calculated because data were essentially constant (all participants attended three times at their gestational week 13)

EXE sessions were delivered six days a week and the participants were recommended to choose three of the sessions (Figs. 1 and 2). During the trial period of approximately 32 months, one EXE session was cancelled due to sickness among the intervention providers. Only during few holiday periods were EXE sessions offered less than six days a week and some sessions were rescheduled. No MOT sessions were cancelled by the intervention providers. A few MOT sessions were scheduled out of range due to holidays or sickness. However, providers strived to reschedule the sessions as close to the allocated period as possible (Figs. 1 and 2). During the process of re-designing the interventions into the online setup due to COVID-19 restrictions, six consecutive EXE sessions were cancelled.

Participants in both intervention groups expressed in the interviews that the intervention accessibility was high. All participants in EXE expressed that the accessibility of the sessions was important to fit the exercise training into their daily lives. Some expressed that the scheduled sessions resulted in a regular exercise routine in which they preferred to attend sessions on the same weekdays. Some participants even scheduled the sessions into their work calendar to indicate to colleagues that they were occupied. For others, the timing of the EXE sessions was a barrier to participation, as it was difficult to fit in to their everyday life and commitments. They were dependent on the frequently offered EXE sessions to devise a more flexible schedule. A woman in EXE mentioned that:

*“It (attending exercise sessions) has been a bit difficult to juggle, but being employed as I am, I have quite flexible working hours, and as the sessions were offered on so many different days, I could sort of choose the days when I didn’t have to show physically for work”* (Participant no. 73, EXE).

### Dose received

Throughout the trial period, participants randomised to EXE attended on average 1.3 [95% confidence interval, 1.1; 1.5] sessions/week of the recommended 3 sessions/week from randomisation to birth. The attendance rate in the online setup of the EXE intervention was 45% higher compared to the attendance rate in the physical setup (online: 1.6 [1.3; 2.0] sessions/week; physical: 1.1 [0.9; 1.4] sessions/week, *p* = 0.027) [18].

During the trial period 28% (*n* = 24) of the 87 participants in EXE participated on average in 2 or more sessions/week, 32% (*n* = 28) participated on average in 1-1.9 sessions/week, and 40% (*n* = 35) participated on average in less than 1 session/week. Among the 48 participants who received the physical EXE intervention only, 19% (*n* = 9) attended 2 or more sessions/week, 35% (*n* = 7) attended 1-1.9 session/week, and 46% (*n* = 22) attended less than 1 session/week. Among the 25 women who received the online EXE intervention only, 52% (*n* = 13) attended 2 or more sessions/week, 24% (*n* = 6) attended 1-1.9 sessions/week and 24% (*n* = 6) attended less than 1 session/week. The attendance rate in EXE in relation to gestational age is presented in Fig. 2.

Dose received among participants in EXE who were still included and not lost to follow-up did not differ from dose received among all participants randomised to EXE. Throughout the trial period, morning and afternoon sessions seemed to be equally attractive, whereas the Saturday session (a morning gym session) was the most attended during the week (Table 3).

**Table 3:** Distribution of sessions attended (number and percentages) of the structured supervised exercise training (EXE) in days of the week, gym or pool, and time of the day. Before COVID-19, from October 2018 to March 11th, 2020; During COVID-19, from March 12th, 2020 to May 2021 due to COVID-19 restrictions. n, number. During the study period participants in EXE overall joined a session 3000 times. Mon, Monday; Tue, Tuesday; Wed, Wednesday; Thu, Thursday; Fri, Friday; Sat, Saturday

	Week days						Type of session		Time of session		Overall
	**Mon**	**Tue**	**Wed**	**Thu**	**Fri**	**Sat**	**Gym**	**Pool**	**Morning**	**Afternoon**	
**Sessions attended, n (%)**											
**The entire trial period**	572 (19)	392 (13)	458 (15)	526 (18)	396 (13)	656 (22)	2226 (74)	774 (26)	1444 (48)	1556 (52)	3000 (100)
**Before COVID-19**	339 (20)	243 (14)	215 (13)	335 (20)	257 (15)	322 (19)	1133 (66)	578 (34)	822 (48)	889 (52)	1711 (57)
**During COVID-19**	233 (18)	149 (12)	243 (19)	191 (15)	139 (11)	334 (26)	1093 (85)	196 (15)	622 (48)	667 (52)	1289 (43)

Throughout the trial period, participants randomised to MOT attended 5.2 [4.7; 5.7] out of 7 counselling sessions (74%) during their pregnancies. The number of MOT sessions attended did not differ between participants offered physical or online sessions (physical: 5.3 [4.6; 6.0]; online: 5.6 [4.8; 6.4], *p* = 0.970) [18]. 64% of the 87 participants in MOT (*n* = 56) attended six or seven sessions, 13% (*n* = 11) attended four or five sessions and 23% (*n* = 20) attended up to three sessions. More than 80% of participants randomised to MOT attended the first group and the first individual session whereas 57% attended the last group session (Table 4).

**Table 4:** Attendance in group and individual sessions in MOT during the trial period. Distribution of the seven counselling sessions in MOT: G1, < 3 weeks after randomisation; I1, 4–6 weeks after randomisation; I2 and I3, equally distributed between I1 and G2; G2, gestational age (GA) 24–26 weeks; I4, GA 31–32 weeks; G3, GA 35–37 weeks. G, group session; I, individual session; n, number

	G1	I1	I2	I3	G2	I4	G3
**Participants, n (%)**	71 (82)	75 (86)	69 (79)	68 (78)	55 (63)	63 (72)	50 (57)

The average percentage of attendance in group and individual sessions were 67% and 79%, respectively. Among participants not lost to follow-up in MOT, more than 80% attended the first group and all individual sessions and approximately 70% attended group session 2 and 3. The average percentage of attendance in group and individual sessions was 76% and 89%, respectively.

In both intervention groups, participants expressed in the interviews that being part of a group was valued, but that it was seen only as a fun and enjoyable factor and not to network or build new relationships. To some degree, participants in EXE expressed that it was difficult to participate in the sessions due to work, logistics, and family commitments in their everyday life, which they to a larger extent than usual needed to organise. They experienced that in relation to some family activities they were less present than they used to be and wanted to be. In addition, they were more dependent than usual on their partner, for example, to pick up and drop off their children at day care or school etc. and accompany them to leisure activities due to scheduled EXE sessions. A participant in EXE, aged 30 years and with a three-year old child, described how she and her husband organised everyday activities:

*“Well, we need to do some planning*. *For example, I often attend [training sessions] on Wednesday afternoons, and my daughter has also started gymnastics - so they (husband and child) also come home late, and we will eat leftovers that day”* (Participant no. 117, EXE).

Furthermore, it was difficult for participants in EXE to take part in family routines such as evening meals or preparation of these on days with an afternoon EXE session. For some participants, this led to a sense of guilt for not being present in family matters. However, participating in EXE sessions was perceived as a good opportunity to focus on oneself and, despite spending less time with the family, the women experienced increased energy to take care of older children and everyday chores at other times. On a purely practical level, participants in EXE expressed that they needed a car to be able to reach the gym or swimming pool. A 31-year-old woman explained how attendance was hindered:

*“I didn’t really think transportation would matter, but it did, because we only have one car … I had to drop off my child beforehand, it just didn’t add up. I actually invested in a travel card for the train, but it was so much easier when the car was available”* (Participant no. 87, EXE).

Commuting back and forth to the EXE sessions was by some of the participants in EXE not living near the training facilities, perceived as time heavy and as a barrier towards participation. In contrast, commuting was expressed as one of the most significant changes in the everyday life among a large part of participants in MOT. Instead of driving between their workplace and home as they normally would, they incorporated physical active commuting like biking. In addition, the participants in MOT incorporated more PA into already existing activities and added new activities that also involved family members. Participants in MOT expressed that it was important for them not to let their PA level limit their presence in family matters. A woman who was unemployed tried to schedule her exercise routines by separating them from family time:

*“I wanted to be physically active while my boyfriend was at work and my daughter was at day care, so in that way I don’t think it (her being physically active) had any impact on our daily lives”* (Participant no. 71, MOT).

In contrast, a woman with two older children combined family time with her being physically active:

*“My children do gymnastics twice a week, and instead of them biking alone, I bike with them. They find it very nice. Additionally, my husband and I have had a few more evening walks together just the two of us while the kids were at home. It was really nice because I’ve also needed to “achieve” some more steps (on the tracker). My husband just said: “Okay, then I’ll come with you””* (Participant no. 109, MOT).

Notably, it seemed like participation in PA in MOT was perceived as easier to fit into everyday life, and that it caused less conflicts in planning everyday life than what was perceived among participants in EXE.

### Mechanisms of impact

In general, participants in both intervention groups expressed in the interviews that they valued the interventions and appreciated being part of a research trial. Some of the participants expressed that they were able to plan their own working hours, which allowed them to participate in the interventions. A mechanism of impact was that the scheduled EXE sessions represented a *commitment* that participants in EXE felt responsible for keeping. It resulted in participants not having to continually “renegotiate”, either with their families or with themselves, to prioritise time for PA in their daily lives. Participants in EXE expressed that having intervention providers and other EXE participants waiting for them influenced highly on their commitment to the intervention and was a motivator for being physically active:

*“I’m a very dutiful person, so when something is in my calendar and I’ve said it’s a deal, well, it’s a deal. I’m not so dutiful when it comes to my own obligations to myself. But when I say I’m going to show up, I show up.“* (Participant no. 73, EXE).

In contrast, participants in MOT expressed that they felt self-determined towards PA and how to structure and organise PA on their own while supervised and supported by the intervention providers. A *perception of empowerment* was one of the most motivating and important mechanisms of impact for participation in MOT and for their PA level and intervention maintenance. As participants in EXE, they expressed a great ability to independently structure their everyday life, which was essential for participation:

*“I have a job where I have a lot of flexibility, so when I had to go in for a counselling session, I’ve just taken time off and worked at another time”* (Participant no. 124, MOT).

Another mechanism of impact was the *perceptions of PA* which were notably different between the two groups. Participants in EXE considered PA to be an event that took place at a specific time point. Once they had participated in an EXE session, PA was not considered integrated in the remaining day:

*“Well, I think (when attending an exercise session), I can tick that one off. Then I have kind of been active today. It was like one of those things that I had on my agenda”* (Participant no. 103, EXE).

It appeared that participants in EXE separated everyday activities from what they perceived as actual exercise and distinguished between PA intensities. They found the sessions to be fruitful and valuable, but at the same time they noted the low degree of autonomy regarding the specific content of the sessions. For example, some of the participants in EXE found the 30 min session on the stationary bike (the first part of the 1-hour session in the original setup) to be monotonous and bland. However, their motivation was that stationary biking was the best activity to increase the heart rate to the required level when they felt heavier which made them continue. As oppose to the understanding of PA as an event in the EXE group, MOT participants seemed to have integrated PA more in daily activities. Participants in MOT expressed that PA of all kinds was considered valid, regardless of intensity. A woman expressed it like this:

*“Exercise doesn’t have to be me going to the gym three times a week or me going for that run like everybody else does. This thing about exercise, it can be many things. It can also be that I just take the stairs 10 times or that I just walk faster with the pram now that I’m out walking anyway. So, I think it (PA) just became more simplified. That it doesn’t have to be so difficult”* (Participant no. 133, MOT).

The different perceptions of PA were also expressed in the way that the participants referred to the mental and physical reactions and changes they experienced. Participants in EXE focused particularly on bodily capacities, changes, and appearance:

*“You can tell from my body that I’ve been training hard … I can see it in my posture and just things like thighs and glutes and arms and stuff. They were maybe just a bit more untrained [before]”* (Participant no. 81, EXE).

and

*“I think it has been amazing to feel that I have become stronger”* (Participant no. 86, EXE).

Participants in MOT expressed a somewhat broader perception of PA effects as they found themselves with greater insight and understanding of themselves being pregnant and with increased mental health and well-being:

*“I’ve really felt good about my body in this pregnancy, and I think that’s so great. I think it’s largely because I’ve gotten to know my body and I’m in such good shape”* (Participant no. 124, MOT)

and

*“I think it (being a part of the intervention) had an impact on my well-being in general, including my mental well-being. Because I can feel my mood gets better, when I exercise* (Participant no. 74, MOT).

## Discussion

This process evaluation demonstrated that the FitMum trial *reached* a selected group of pregnant women with a high educational level. Compared with the general population of pregnant women who delivered at Copenhagen University Hospital - North Zealand in 2017, participants in the FitMum trial were more likely to have an educational level at or above a bachelor’s degree (80% vs. 29%) (data not shown). A higher educational level might have led to more flexibility in working and everyday life although it per protocol was aimed to include participants representative for the general pregnant Danish population [17]. The participants had altruistic and personal reasons to participate in the FitMum trial and a general interest in their own and their unborn child’s health, which in line with previous studies, enabled the prioritisation of PA in their everyday life [10, 32]. Notably, the participants perceived being physically active as a prerequisite to an uncomplicated pregnancy and delivery and expressed a potential self-blame if any complications occurred. A paradox arose between a desire for being physically active and a sense of guilt for spending less time with their family. It was identified that the interventions (*dose*) were well delivered, and that the implementation *fidelity* was high in both interventions applied in the original setup with physical attendance and in the altered, online setup implemented during the COVID-19 pandemic. A low and varying *dose* of the EXE intervention received, especially in the physical setup, might be explained by the fact that the attendance in EXE relied on high everyday flexibility among participants. During the COVID-19 restrictions, everyday life changed radically with positive consequences for the dose received among participants in EXE. The high intervention accessibility was important for the participants to adapt PA into their everyday life. Baseline characteristics of the participants and their high everyday autonomy influenced the *dose* received in EXE more than anticipated and thus provided insights into unanticipated contextual factors. It could be hypothesised that barriers addressed in the resent process evaluation more likely could be accommodated rather than intrapersonal barriers as fatigue, discomforts, and uncertainty about exercising as reported by several other studies.

This process evaluation showed that the interventions and the way participants were influenced by them affected the *mechanisms of impact* differently in the two intervention groups. This was reflected, among other things, in a discrepancy in the perception of PA among participants in the two intervention groups. With the Self-Determination Theory [33] as a theoretical basis, the approach was that behaviour is complex and that people are rarely driven by either intrinsic or extrinsic motivation. Behaviour often tends to lie in the middle of either pure self-determination driven by pleasure and interest (intrinsic) and at the other end of the continuum the non-self-determined behaviour performed out of necessity or obligation (extrinsic). In brief, the theory posits that people are driven by a connection to others, competence to perform a given task, and autonomy of one’s behaviour to achieve psychological growth. As an example, participants in EXE experienced an extrinsic motivation because of the experienced commitment to the intervention, which fuelled an intrinsic motivation supported by the experienced ability to develop a routine for attending the sessions as well as the perceived bodily changes. As fatigue and lack of time are commonly cited barriers towards PA in pregnancy, participation in structured exercise may improve general PA behaviour [10]. Participants in EXE felt a *connection* to the intervention whereas participants in MOT expressed a high *perceived competence and autonomy* towards PA, expressed as intrinsic motivators such as the high *perception of empowerment* towards PA. Conversely, extrinsic motivators including *commitment* to others and to the trial itself were expressed by participants in both interventions.

A systematic review [22] on issues of internal and external validity in interventions to improve PA during pregnancy found that reach and efficacy of the interventions were well reported in randomised controlled trials and quasi-experimental studies with a comparator group included. However, information on for example dose, representativeness of participants and setting were less commonly reported. To the best of our knowledge, few process evaluations of PA interventions during pregnancy have been performed, and none of them had a scope directly comparable to the present trial [23, 24]. The process evaluations of the pilot study of Vitamin D and Lifestyle Intervention (DALI) [23] and the UK Pregnancies Better Eating and Activity Trial (UPBEAT) [24] focused on lifestyle interventions including PA to prevent gestational diabetes mellitus among overweight and obese pregnant women. Findings from the process evaluations of these studies [23, 24] coincided with some of the findings in the present trial and revealed that practicalities often interfered with regular attendance in sessions even though participants claimed that they were willing to attend. Moreover, the DALI study revealed that participation was very time-consuming for the women, which led to lower participation rates [23]. Few effect evaluations of interventions comparable to the FitMum trial included process evaluation dimensions such as reach and dose. Those dimensions are often reported in connection with the presentation of participant flowcharts and attendance rates [15, 16, 34, 35]. Wang et al. [35] reported a high attendance rate in a gym-based intervention, however, no mechanisms of impact were reported except from a high predefined limit of intervention adherence that might have influenced the attendance. In contrast, Oostdam et al. [16] reported a low attendance rate in a gym-based intervention which was to some degree explained by low intervention accessibility. For future perspectives the mechanisms of impact as commitment, perception of empowerment and perception of PA as well as the paradox between prioritising PA and family and the need of a flexible everyday life need to be considered. When implementing PA interventions, the “efficacy paradox” should be paid attention [36]. This means that an effective intervention, when studied under optimal conditions, might not be as effective when applied in a real-world setting. Less effective interventions may have a greater potential of implementation in people’s everyday life and environments.

### Strength and limitations

The main strength of the trial was the application of a mixed methods design [25], which provided a comprehensive insight into how the two complex PA interventions were implemented and an explanatory interpretation of how they produced changes. The application of the Medical Research Council process evaluation framework [20] enabled us to report findings of the process evaluation dimensions and to illustrate facilitators and barriers influencing the intervention implementation and efficacy. Moreover, the framework supported an understanding of context and potential mechanisms of impact related to the effects of the FitMum trial on PA. A limitation was that the unintended alterations of the intervention design due to the COVID-19 restrictions were only quantitatively covered. While the attendance rate in EXE was significantly higher in the online intervention compared to the physical, a qualitative insight investigating any reasons could provide important knowledge. Another limitation was that process evaluation data were among others collected by one of the intervention providers (SdPK). However, this person’s in-depth knowledge of the structure and content of the interventions supported a comprehensive process evaluation.

## Conclusion

This mixed methods process evaluation demonstrated that participants *reached* in the FitMum trial had a higher everyday life autonomy and educational level compared to the general population of pregnant women. The PA interventions (*dose*) were well *delivered* with high *fidelity* in the original physical intervention setup as well as in the altered online intervention setup delivered during the COVID-19 restrictions. Although intervention accessibility was expressed as high, the *dose received* in EXE were low and varying. During the online EXE setup, the dose received increased compared to the physical EXE setup. This may be explained by competing interests between spending time on PA and family life. *Mechanisms of impact* comprised among participants in EXE a commitment to the intervention and flexible everyday life, whereas perception of empowerment towards PA was essential among participants in MOT. The perception of PA was different in the two intervention groups as participants in EXE considered PA to be a time constrained activity, whereas participants in MOT thought of PA as everyday activities with paying less attention to PA intensity. For future perspectives, prenatal PA interventions might benefit from integrating a combination of physical attendance at e.g. one-hour structured supervised exercise sessions and frequent 30-min home-based, online supervised exercise sessions to increase the dose received among the pregnant women. In addition, an awareness of PA perception, PA empowerment and commitment to others should be considered.

## Supplementary Information

**Additional file 1.** Interview guide.

## Data Availability

The Danish Data Protection Agency has not approved data sharing. The datasets used in the current trial are available from the corresponding author on reasonable request.

## Abbreviations

CON: Standard care
EXE: Structured supervised exercise training
Min: Minutes
MOT: Motivational counselling on physical activity
MVPA: Moderate-to-vigorous-intensity physical activity
PA: Physical activity

## References

[1] Fair F, Soltani H. A meta-review of systematic reviews of lifestyle interventions for reducing gestational weight gain in women with overweight or obesity. Obesity Reviews. 2021;1–21. 10.1111/obr.13199. 10.1111/obr.13199 PMC8047893 33459493

[2] Ruchat SM, Mottola MF, Skow RJ, Nagpal TS, Meah VL, James M (2018). Effectiveness of exercise interventions in the prevention of excessive gestational weight gain and postpartum weight retention: a systematic review and meta-analysis. Br J Sports Med.

[3] Díaz-Burrueco JR, Cano-Ibáñez N, Martín-Peláez S, Khan KS, Amezcua-Prieto C (2021). Effects on the maternal-fetal health outcomes of various physical activity types in healthy pregnant women. A systematic review and meta-analysis. Eur J Obstet Gynecol Reprod Biol.

[4] Hayes L, McParlin C, Azevedo L, Jones D, Newham J, Olajide J (2021). The effectiveness of Smoking Cessation, Alcohol Reduction, Diet and physical activity interventions in improving maternal and Infant Health Outcomes: a systematic review of Meta-analyses. Nutrients.

[5] Mottola MF, Davenport MH, Ruchat SM, Davies GA, Poitras VJ, Gray CE (2018). 2019 canadian guideline for physical activity throughout pregnancy. Br J Sports Med.

[6] Physical Activity Guidelines Advisory Committee Scientific Report. 2018 Physical Activity Guidelines Advisory Committee. Washington, DC: US Department of Health and Human Services. 2018;(F8-1).

[7] Bhattacharjee J, Mohammad S, Adamo KB (2021). Does exercise during pregnancy impact organs or structures of the maternal-fetal interface?. Tissue Cell.

[8] Morales-Suárez-Varela M, Clemente-Bosch E, Peraita-Costa I, Llopis-Morales A, Martínez I, Llopis-González A. Maternal physical activity during pregnancy and the Effect on the Mother and Newborn: a systematic review. J Physical Activity Health. 2020;18(1):1–18. 10.1123/jpah.2019-0348. 10.1123/jpah.2019-0348 33361475

[9] Sánchez-Polán M, Franco E, Silva-José C, Gil-Ares J, Pérez-Tejero J, Barakat R (2021). Exercise during pregnancy and prenatal depression: a systematic review and Meta-analysis. Front Physiol.

[10] Harrison AL, Taylor NF, Shields N, Frawley HC (2018). Attitudes, barriers and enablers to physical activity in pregnant women: a systematic review. J Physiotherapy.

[11] Coll CVN, Domingues MR, Gonçalves H, Bertoldi AD (2017). Perceived barriers to leisure-time physical activity during pregnancy: a literature review of quantitative and qualitative evidence. J Sci Med Sport.

[12] Oude Rengerink K, Logtenberg S, Hooft L, Bossuyt PM, Mol BW. Pregnant womens’ concerns when invited to a randomized trial: a qualitative case control study. BMC Pregnancy Childbirth. 2015;15(1):207. 10.1186/s12884-015-0641-x PMC4560072 26341516

[13] Pearce EE, Evenson KR, Downs DS, Steckler A. Strategies to promote physical activity during pregnancy. Am J Lifestyle Med. 2013;7:38–50. 10.1177/1559827612446416 PMC3866032 24363633

[14] James P, Morgant R, Merviel P, Saraux A, Giroux-Metges MA, Guillodo Y, et al. How to promote physical activity during pregnancy: a systematic review. Journal of Gynecology Obstetr Human Reprod. 2020;49(9). 10.1016/j.jogoh.2020.101864. 10.1016/j.jogoh.2020.101864 32663651

[15] Seneviratne SN, Jiang Y, Derraik JGB, McCowan LME, Parry GK, Biggs JB (2016). Effects of antenatal exercise in overweight and obese pregnant women on maternal and perinatal outcomes: a randomised controlled trial. BJOG.

[16] Oostdam N, Van Poppel MNM, Wouters MGAJ, Eekhoff EMW, Bekedam DJ, Kuchenbecker WKH (2012). No effect of the FitFor2 exercise programme on blood glucose, insulin sensitivity, and birthweight in pregnant women who were overweight and at risk for gestational diabetes: results of a randomised controlled trial. BJOG.

[17] Roland CB, Knudsen S, de Alomairah P, Andersen SA, Bendix AD, Clausen J (2021). Structured supervised exercise training or motivational counselling during pregnancy on physical activity level and health of mother and offspring: FitMum study protocol. BMJ Open.

[18] Knudsen SDP, Alomairah SA, Roland CB, Jessen AD, Hergel I-M, Clausen TD (2022). Effects of structured supervised Exercise training or motivational counseling on pregnant women’s physical activity level: FitMum Randomized Controlled Trial. J Med Internet Res.

[19] Bull FC, Al-Ansari SS, Biddle S, Borodulin K, Buman MP, Cardon G, et al. World Health Organization 2020 guidelines on physical activity and sedentary behaviour. Brit J Sports Med. 2020;54:1451–62. 10.1136/bjsports-2020-102955 PMC7719906 33239350

[20] Moore G, et al. Process evaluation of complex interventions. Medical Research Council guidance; 2015. 10.1136/bmj.h1258 PMC4366184 25791983

[21] Skivington K, Matthews L, Simpson SA, Craig P, Baird J, Blazeby JM, et al. A new framework for developing and evaluating complex interventions: update of Medical Research Council guidance. BMJ. 2021;374:n2061. 10.1136/bmj.n2061. 10.1136/bmj.n2061 PMC8482308 34593508

[22] Craike M, Hill B, Gaskin CJ, Skouteris H (2017). Interventions to improve physical activity during pregnancy: a systematic review on issues of internal and external validity using the RE-AIM framework. BJOG.

[23] Jelsma JGM, Simmons D, Gobat N, Rollnick S, Blumska K, Jans G (2017). Is a motivational interviewing based lifestyle intervention for obese pregnant women across Europe implemented as planned? Process evaluation of the DALI study. BMC Pregnancy Childbirth.

[24] Poston L, Briley AL, Barr S, Bell R, Croker H, Coxon K (2013). Developing a complex intervention for diet and activity behaviour change in obese pregnant women (the UPBEAT trial); assessment of behavioural change and process evaluation in a pilot randomised controlled trial. BMC Pregnancy Childbirth.

[25] Creswell JW, Clark VL (2017). Designing & conducting mixed methods research + the mixed methods reader. Designing & conducting mixed methods research + the mixed methods reader.

[26] Kvale S, Brinkmann S. Interview: det kvalitative forskningsinterview som håndværk. Kvale S, Brinkmann S, editors. Hans Reitzels Forlag; 2015.

[27] Othman S, Steen M, Fleet J-A (2020). A sequential explanatory mixed methods study design: an example of how to integrate data in a midwifery research project. J Nurs Educ Pract.

[28] Fetters MD, Curry LA, Creswell JW (2013). Achieving integration in mixed methods designs - principles and practices. Health Serv Res.

[29] QSR International Pty Ltd., NVivo. 2020.

[30] Malterud K. Kvalitative forskningsmetoder for medisin og helsefag. Universitetsforlaget; 2017.

[31] Min-Mave.dk. [cited 2022 Jul 1]. Available from: https://min-mave.dk/.

[32] Rockliffe L, Peters S, Heazell AEP, Smith DM (2021). Factors influencing health behaviour change during pregnancy: a systematic review and meta-synthesis. Health Psychol Rev.

[33] Ryan R, Deci E (2000). Self-determination theory and the facilitation of intrinsic motivation. Am Psychol.

[34] Leung Hui A, Back L, Ludwig S, Gardiner P, Sevenhuysen G, Dean HJ (2014). Effects of lifestyle intervention on dietary intake, physical activity level, and gestational weight gain in pregnant women with different pre-pregnancy body Mass Index in a randomized control trial. BMC Pregnancy Childbirth.

[35] Wang C, Wei Y, Zhang X, Zhang Y, Xu Q, Sun Y, et al. A randomized clinical trial of exercise during pregnancy to prevent gestational diabetes mellitus and improve pregnancy outcome in overweight and obese pregnant women. Am J Obstetr Gynecol. 2017;216(4):1–30. 10.1016/j.ajog.2017.01.037 28161306

[36] Zhang W, Doherty M (2018). Efficacy paradox and proportional contextual effect (PCE). Clin Immunol.

