# Supplementary material for: Physical activity in pregnancy: a mixed methods process evaluation of the FitMum randomised controlled trial interventions
Source: BMC Public Health. 2022 Dec 6;22:2283. doi: 10.1186/s12889-022-14717-1 (PMC9724308; doi:10.1186/s12889-022-14717-1)
Supplement: Supplementary file 1 — Additional file 1. Interview guide. [file 12889_2022_14717_MOESM1_ESM.docx]

# Supplementary file 1:

# Interview guide

**Inclusion and participation in the FitMum trial:**

When you were enrolled in the FitMum trial, you were asked why you would like to participate. Try to answer the same questions now (no matter your previous answer).

Can you tell me about your thoughts on health and physical activity during pregnancy?

Why and for whom was and is it important to participate?

Did you discuss your participation with your partner or others before you made the decision to attend? If so, what impact did their attitude have on your participation?

What was it like for you to sign up for a project that was only for women who exercised less than one hour a week?

What thoughts did you have before and during participation about challenges/barriers to participation?

Can you tell me about the specific reactions you received from others (partner, family, friends, colleagues) about your participation?

How were you greeted by the FitMum staff when you were enrolled and during the subsequent visits? What influence did this have on your participation?

Please describe any changes to you and your family's everyday life (mainly in terms of physical activity and time used) as a result of your participation in the FitMum trial.

**Perception of the content in the structured supervised exercise training intervention (EXE) or motivational counselling on physical activity intervention (MOT), physical activity in everyday life, and barriers and enablers towards physical activity:**

Please describe a typical day and week while being a part of the FitMum trial.

Please describe a typical day when you attended an intervention session (concrete examples of what you did to participate).

Please describe an intervention session. How did the session proceed, and what was your perception of the session?

What did you do specifically to make it possible for you to participate? (work, family, cooking, shopping, who helped you and how)

What barriers did you perceive to participating (intervention accessibility, content, etc.)?

What could have made it easier to participate?

Please describe your thoughts and perceptions about physical activity in your everyday life. Is there a difference from before pregnancy to now?

In concrete terms, what have you gained from being a part of the structured supervised exercise training group?

**Importance of physical activity during pregnancy:**

Why/why not did you exercise during your present pregnancy?

What motivated you to be physically active?

Did your motivation towards physical activity change during your enrolment in the FitMum trial? Please describe how.

What is important in your everyday life for you to be able to be physically active? (family, support, time spend, leisure time, etc.)

How did your participation in the FitMum trial influence your physical activity level?

Please describe hypothetically how your everyday life (in terms of physical activity) would have been if you had not been enrolled in the FitMum trial.

Do you have anything to add? Thank you very much
